# Supplementary figures and images for: Spatiotemporal dynamics and selectivity of mRNA translation during mouse pre-implantation development
Source: bioRxiv. 2024 Oct 28:2024.10.28.620693. Preprint. [Version 1] doi: 10.1101/2024.10.28.620693 (PMC11565823; doi:10.1101/2024.10.28.620693)

Fig. S1

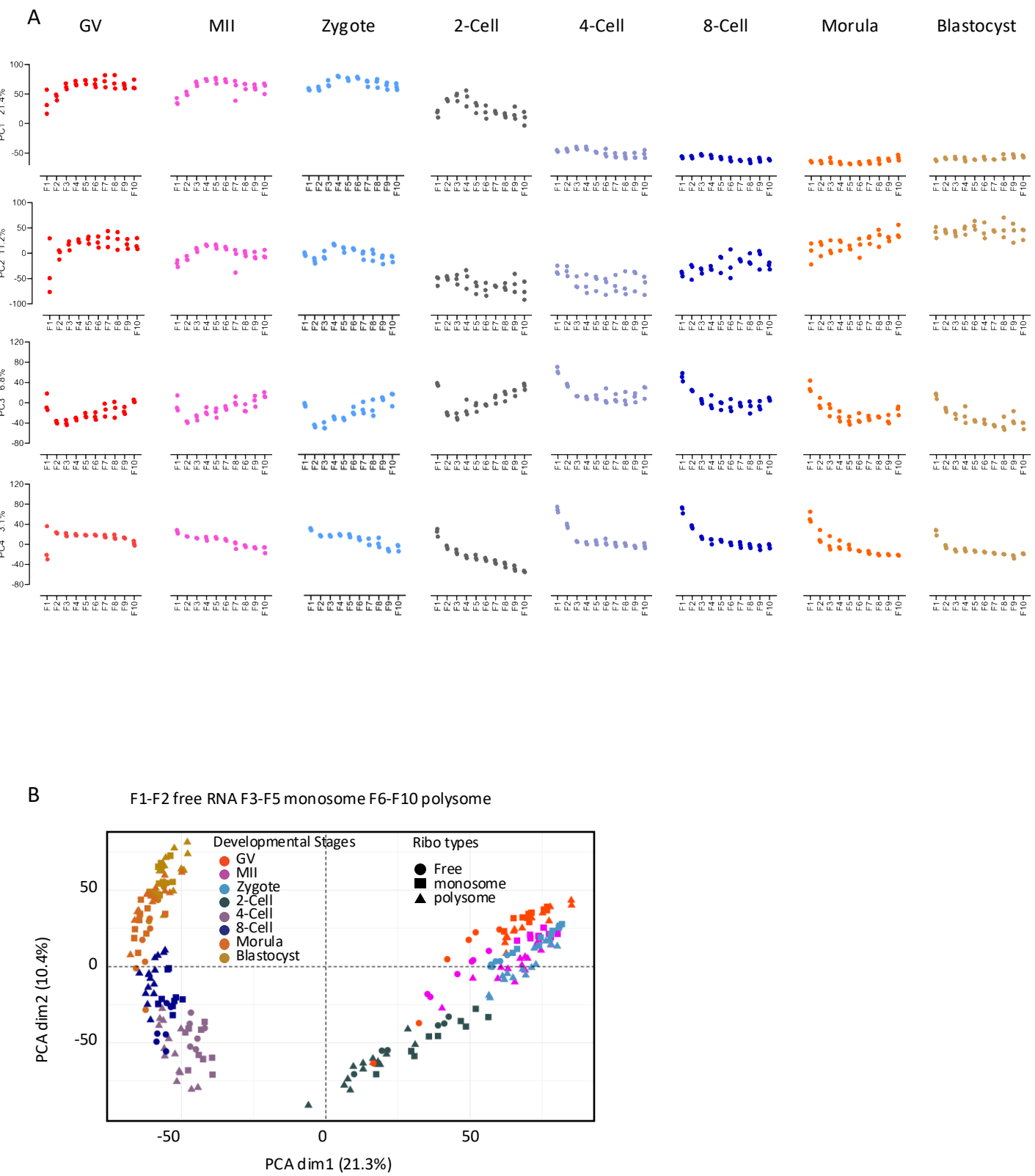

Supplement: Supplement 1 — Figure S1. Principal Component Analysis (PCA) of sequencing data. A. PCA of 4 dimensions (PC1-4) of 10 fractions of mouse oocytes and early embryos. B. Principal Component Analysis (PCA) of free-, monosome- and polysome-bound mRNA profiles in 10 fractions of mouse oocytes and early embryos. [file media-1.pdf]

Fig. S2

A

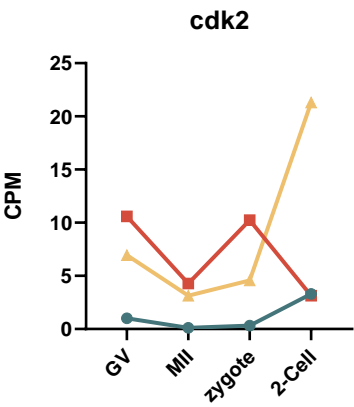

B

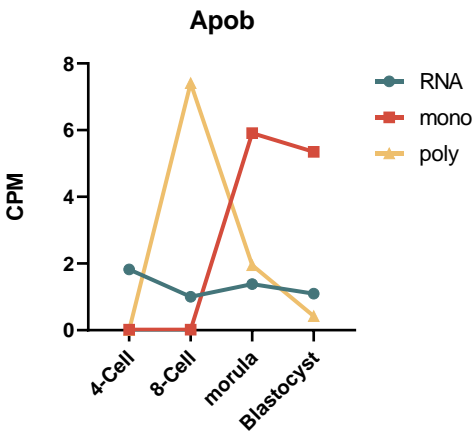

C

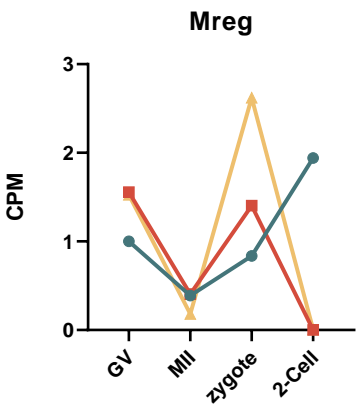

D

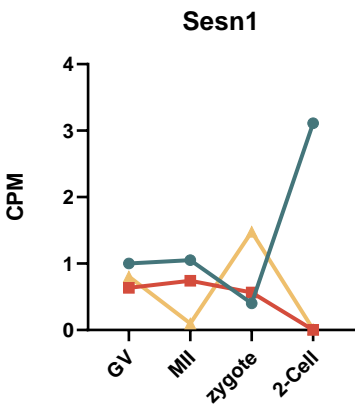

Supplement: Supplement 2 — Figure S2. Flow of genes across modes during mouse oocyte and preimplantation development. A-D. Expression dynamic of candidate mRNAs from polysome profile, monosome profile, and transcriptome profile. [file media-2.pdf]

Fig. S3

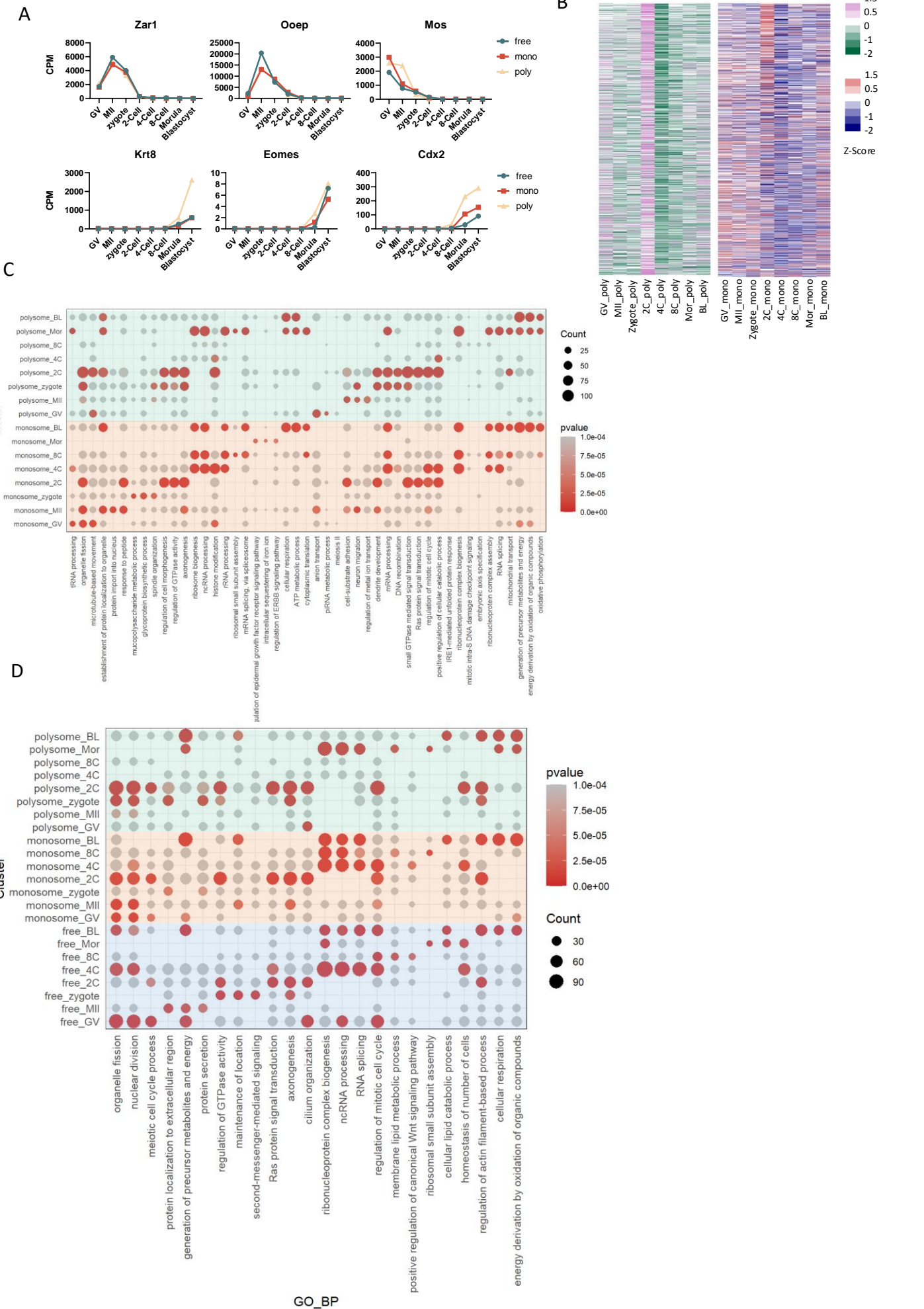

Supplement: Supplement 3 — Figure S3. Comparison of ribosome-bound and ribosome-unbound mRNA in oocytes and early embryos. A. Expression dynamic of selected genes from polysome profile, monosome profile, and free RNA profile. B. Heatmap in both polysome profile and monosome profile showing the expression level of genes from cluster7 in Figure 2A. The color spectrum, ranging from red through white to blue or from green through green to pink, indicates high to low levels of gene expression. C. Dot plot comparison showing top GO terms enriched from stage-specific genes from monosome and polysome datasets. Color from grey to red represents the p-value from high to low, size from small to large represents gene number from low to high. D. Dot plot comparison showing top GO terms enriched from stage-specific genes from free RNA datasets. The color from grey to red represents the p-value from high to low, dots from small to large represents gene number. [file media-3.pdf]

Fig. S4

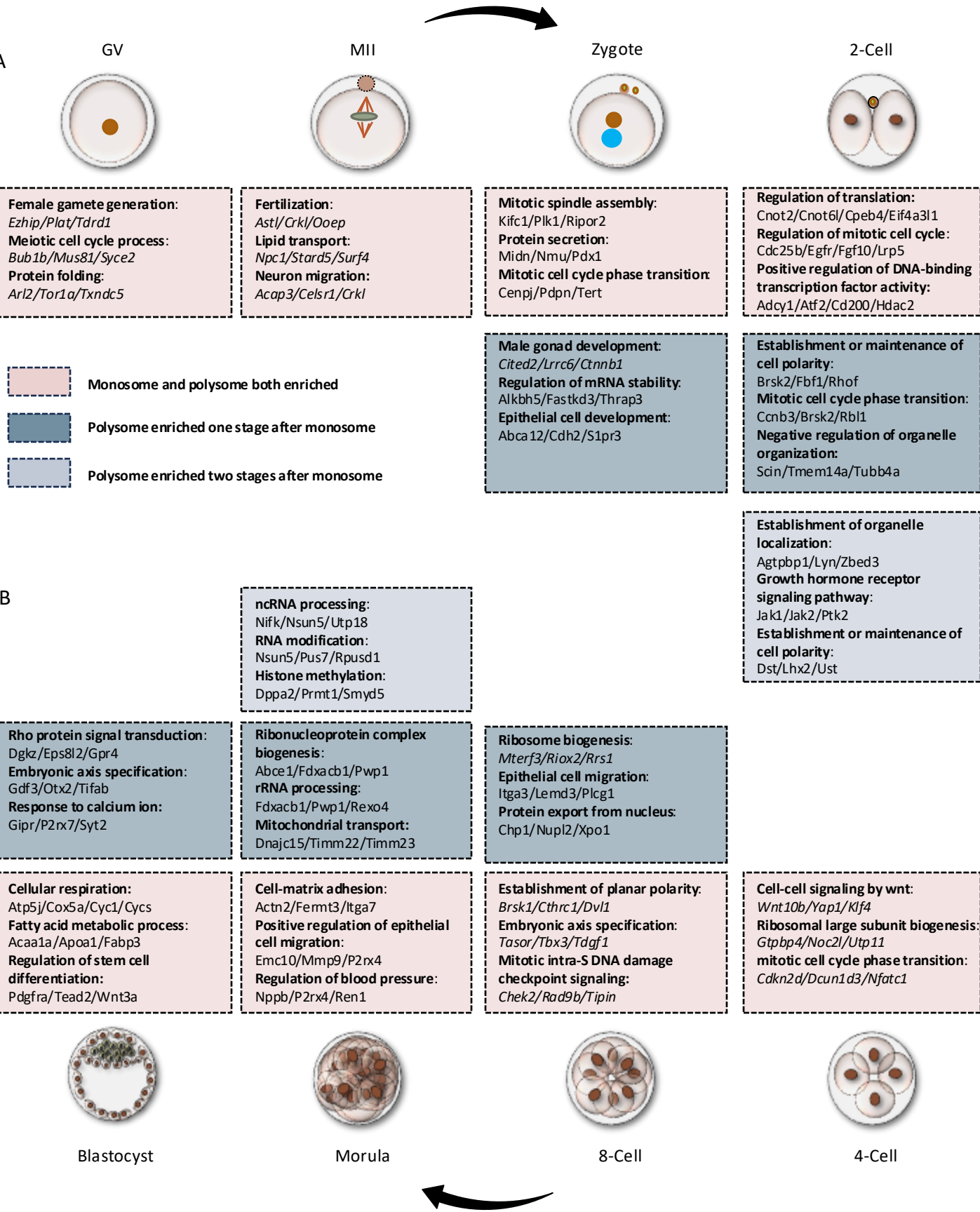

Supplement: Supplement 4 — Figure S4. Trajectory of ribosome bound mRNA during mouse oocyte and preimplantation development. A. Representative overlapped genes peaking from GV till 2-Cell stage from monosome-bound and polysome-bound profiles, as well as the biological functions being regulated correspondingly. B. Representative overlapped genes peaking from 4-Cell till blastocyst stage from monosome-bound and polysome-bound profiles, as well as the biological functions being regulated correspondingly. [file media-4.pdf]

Fig. S5

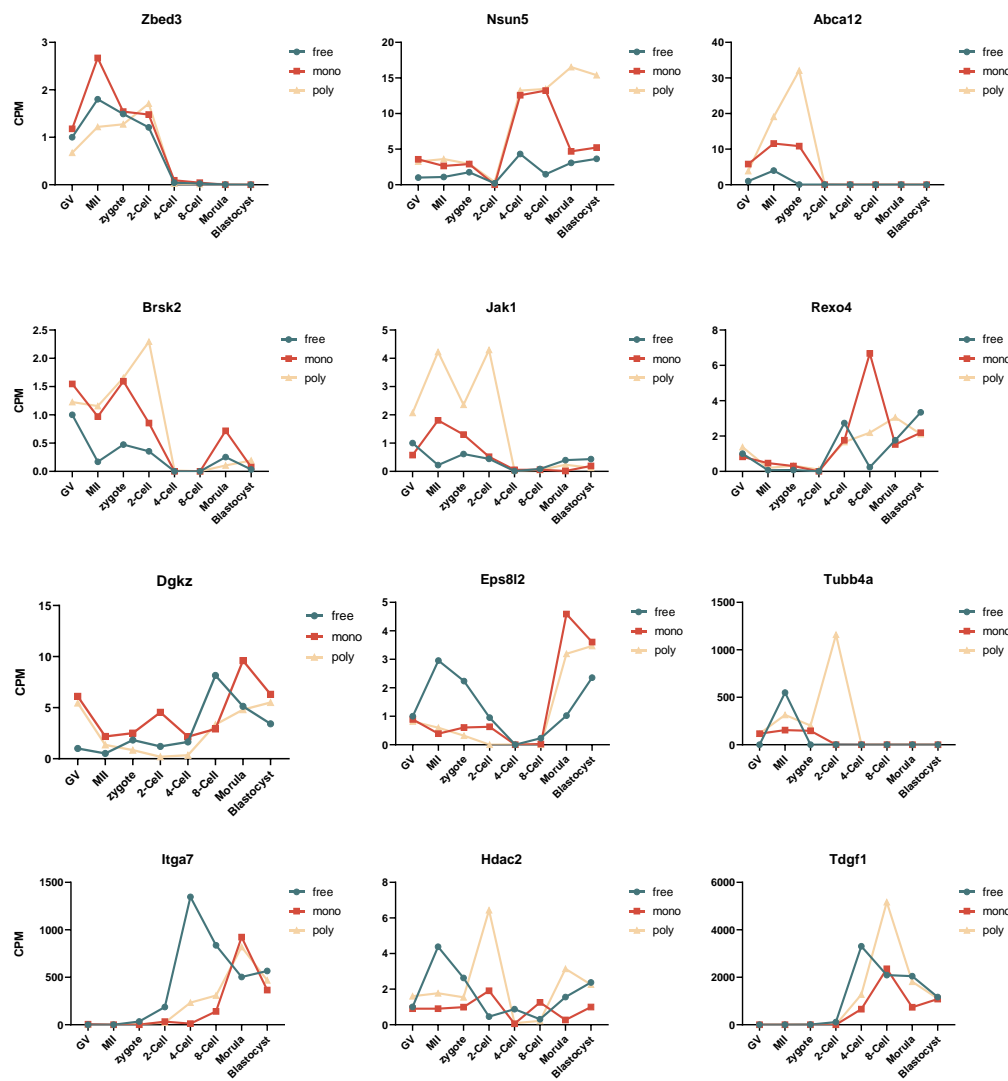

Supplement: Supplement 5 — Figure S5. Expression dynamic of candidate mRNAs from polysome profile, monosome profile, and free RNA profile. [file media-5.pdf]

Fig. S6

GV to MII

Zygote to 2-Cell

A

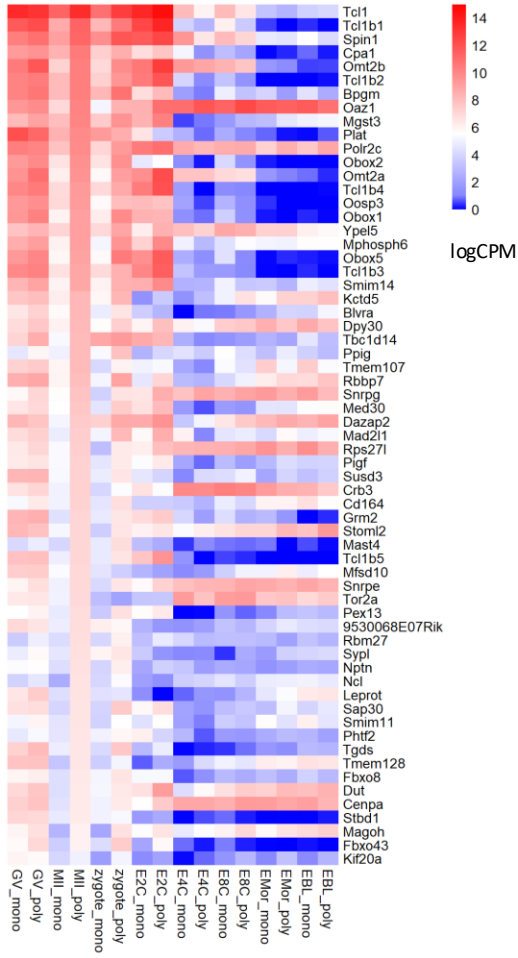

B

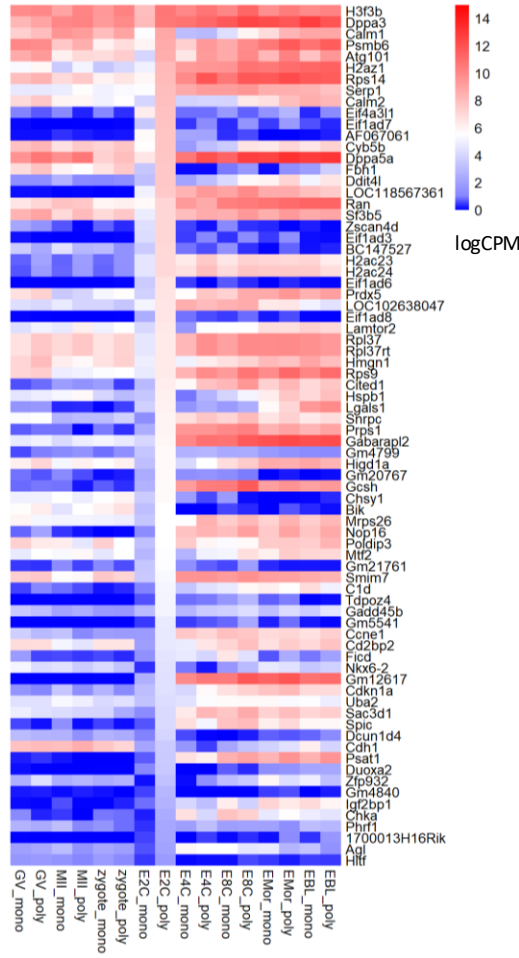

C

8Cell to Blastocyst

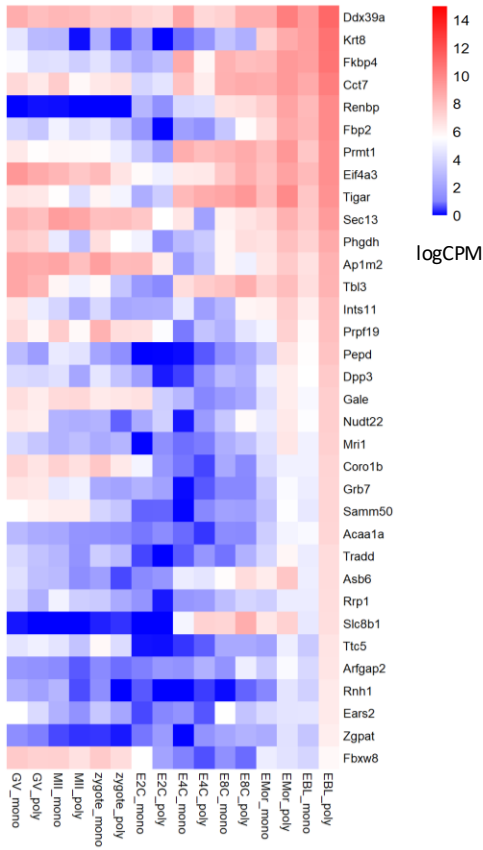

Supplement: Supplement 6 — Figure S6. Genes essential for critical fate transitions during mouse oocyte and early embryo development. A. Heatmap of critical genes translationally activated in GV to MII transition. B. Heatmap of critical genes translationally activated in zygote to 2-Cell transition. C. Heatmap of critical genes translationally activated in 8-Cell to blastocyst transition. The color spectrum, ranging from red through white to blue or from green through green to pink, indicates high to low levels of gene expression. [file media-6.pdf]

Fig. S7

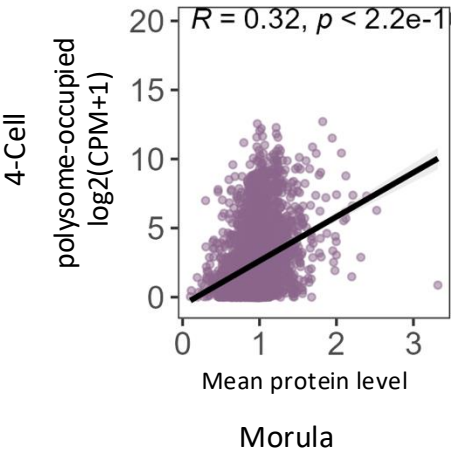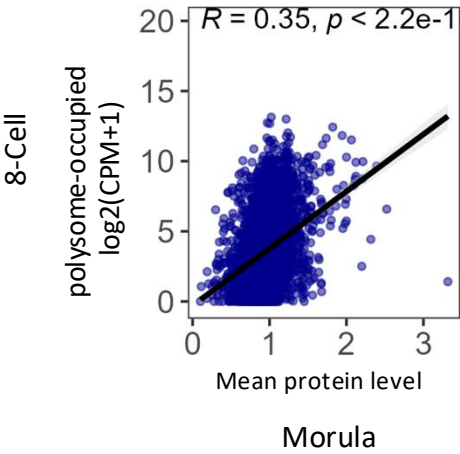

Supplement: Supplement 7 — Figure S7. Correlation between polysome-occupied RNA level (4-/8-Cell) and protein level (morula). [file media-7.pdf]

Fig. S8

A

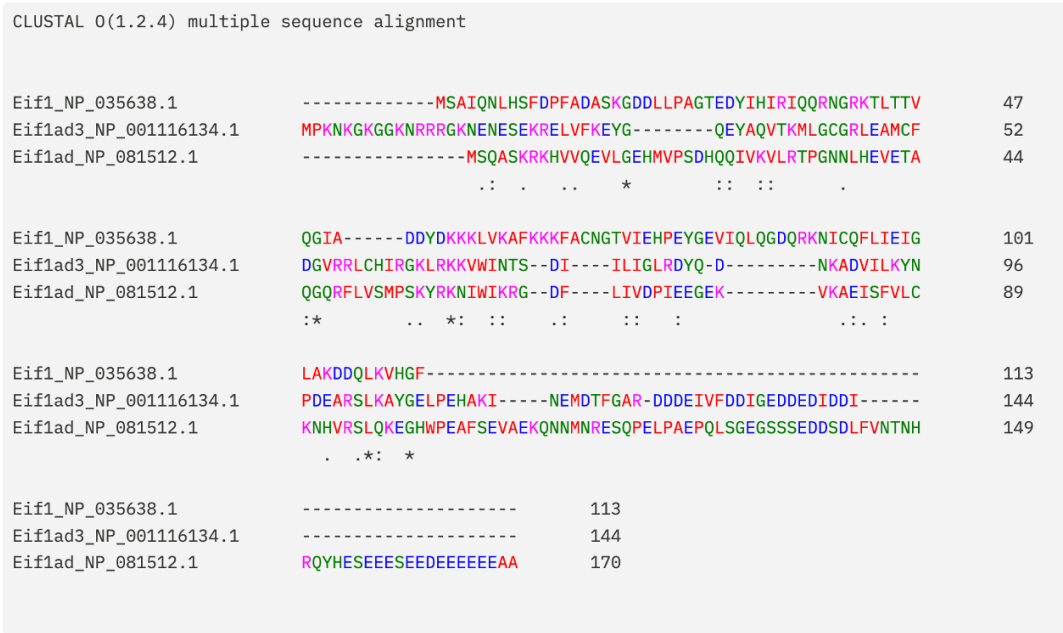

B

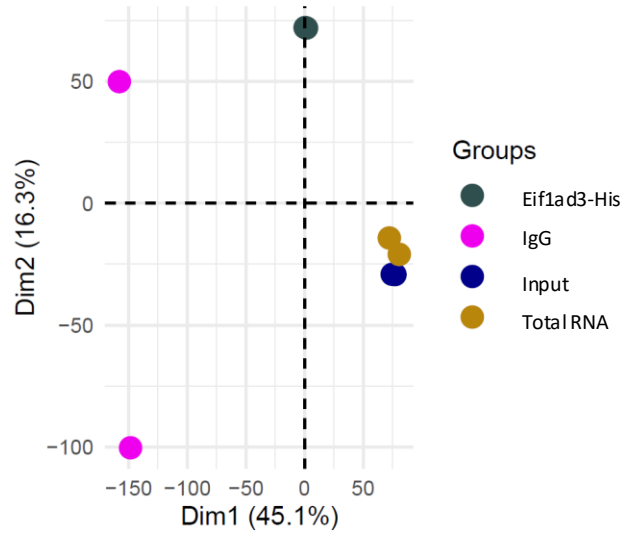

C

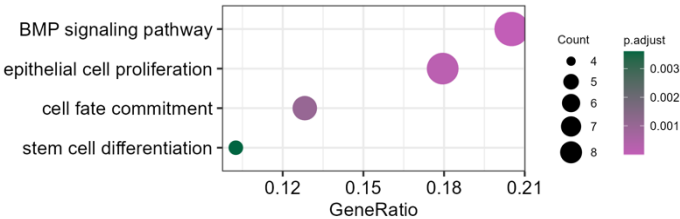

Supplement: Supplement 8 — Figure S8. Injecting Eif1ad3-His into mouse zygote does not affect embryo development. A. Schematic representation of Eif1a, Eif1ad, and Eif1ad3 amino acid sequence. B. PCA of samples of Eif1ad3-His, IgG, negative control, and total RNA groups from RIP-seq. C. Representative GO terms enriched from the transcription factors. Color from pink to green represents the p-value from low to high, size from small to large represents gene number from low to high. [file media-8.pdf]
